# Extracellular Vesicle–Lipid Hybrid Systems for RNA Delivery in Cancer: Structural Classification, Functional Delivery, and Translational Challenges

**DOI:** 10.3390/pharmaceutics18091108

**Published:** 2026-09-02

**Authors:** Lu Lu, Yige Qiu, Jiayu Wu, Wei Dou, Jing Yang, Bo Zhang

**Affiliations:** 1 Department of Pharmacy & State Key Laboratory of Complex Severe and Rare Disease, Peking Union Medical College Hospital, Chinese Academy of Medical Sciences & Peking Union Medical College, Beijing 100730, China; ilulumail@126.com (L.L.); yigeqiu0103@163.com (Y.Q.); douwei000920@163.com (W.D.); 2 State Key Laboratory of Advanced Drug Delivery and Release Systems, Yantai 264003, China; 3 State Key Laboratory of Bioactive Substance and Function of Natural Medicines, Institute of Materia Medica, Chinese Academy of Medical Sciences & Peking Union Medical College, Beijing 100050, China; wujiayu@imm.ac.cn; 4 Bioinformatics Center of AMMS, Beijing 100850, China

**Keywords:** extracellular vesicles, lipid nanoparticles, EV–lipid hybrid systems, RNA delivery, cancer therapy, tumor immunotherapy, intracellular trafficking, safety evaluation

## Abstract

RNA therapeutics offer considerable potential for cancer treatment. Their therapeutic application, however, remains limited by rapid degradation, inefficient cellular uptake, and restricted intracellular release. Extracellular vesicles (EVs) are cell-derived membrane vesicles that have been exploited as promising vehicles for drug delivery due to their high biocompatibility and low immunogenicity, whereas liposomes and lipid nanoparticles provide tunable lipid composition and efficient loading of exogenous nucleic acids. Combining these carriers has led to EV–lipid hybrid systems designed to integrate their complementary properties. This review summarizes recent advances in EV–lipid hybrids for cancer therapy and organizes the reported systems according to their structural architecture and preparation. EV–liposome fusion hybrids, EV–lipid nanoparticle hybrids, and EV membrane-integrated lipid nanocarriers are discussed in relation to their RNA-loading strategies and representative therapeutic designs. The review also examines the key processes involved in functional RNA delivery and summarizes representative applications across different cancer types. Challenges associated with safety evaluation are also discussed, together with future directions for clinical translation. Overall, EV–lipid hybrids represent a promising strategy for RNA-based cancer therapy.

## 1. Introduction

RNA therapeutics, including small interfering RNA (siRNA), microRNA (miRNA), and messenger RNA (mRNA), have attracted increasing attention in oncology [1,2,3]. However, the clinical application of RNA is limited by the intrinsic instability of nucleic acid molecules and delivery barriers. Naked RNA molecules are rapidly degraded by serum nucleases in physiological environments, resulting in a short half-life. In addition, because RNA molecules are negatively charged and hydrophilic, they do not readily cross cellular membranes or efficiently enter the cytosol of target cells. After systemic administration, RNA molecules also lack intrinsic tumor selectivity, which may lead to accumulation in non-target organs, off-target effects, and potential toxicity. Therefore, these delivery barriers remain a major bottleneck [4,5,6]. In recent years, nanocarrier-based delivery platforms, including lipid-based nanocarriers such as lipid nanoparticles, have made significant progress in RNA delivery. However, synthetic lipid carriers may show substantial uptake by clearance organs and limited tumor-cell selectivity [7]. Lipid composition and repeat dosing can also affect immune responses and tolerability [8].

Extracellular vesicles (EVs) are a heterogeneous group of membrane-bound particles released by cells, including small EVs and larger vesicle populations that differ in biogenesis, size, and molecular composition [9,10]. EVs carry proteins, lipids, and nucleic acids and participate in intercellular communication. These properties have prompted their investigation as RNA delivery carriers [11,12].

EV–lipid hybrid systems combine EV-derived membranes with liposomes, lipid nanoparticles, or related lipid structures through fusion, membrane reconstruction, or lipid insertion, and have been explored for RNA delivery and tumor localization [13,14,15,16]. EV–lipid hybrid systems are being investigated because combining EV-derived membranes with synthetic lipids may modify RNA loading, cellular interactions, intracellular transport, and biodistribution. Whether these changes improve cargo-specific RNA activity or antitumor efficacy compared with the parent formulations remains unclear. This review summarizes the reported EV–lipid hybrid systems and their applications in RNA-based cancer therapy. We first compare the properties and limitations of EVs and lipid-based nanocarriers, and then classify the reported hybrid systems according to their structural organization and preparation. Subsequent sections discuss RNA loading and protection, cellular uptake and intracellular transport, and the evidence used to confirm functional RNA delivery. Cancer applications are considered according to tumor type, followed by a discussion of safety concerns and the principal challenges to clinical translation.

## 2. Extracellular Vesicles and Lipid-Based Nanocarriers in RNA Delivery

### 2.1. Extracellular Vesicles as Biologically Derived RNA Carriers

**Figure 1:**
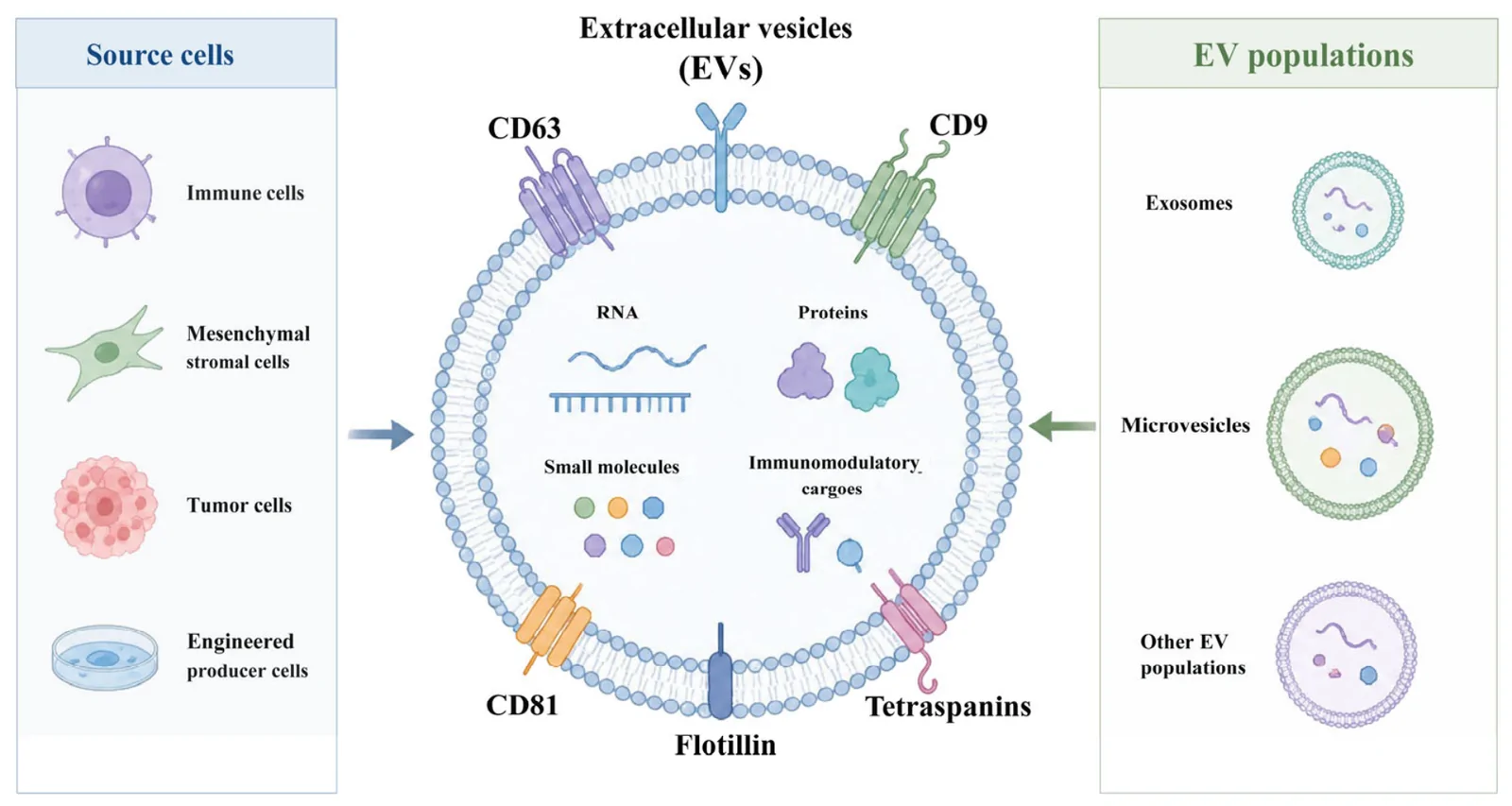
Key features of EVs. EVs are released by diverse source cells, including immune cells, mesenchymal stromal cells, tumor cells, and engineered producer cells. They include exosomes, microvesicles, and other vesicle populations. Representative membrane-associated proteins include the tetraspanins CD9, CD63, and CD81, integrins, and flotillin. Their lipid bilayer, membrane-associated proteins, and cargoes may protect RNA and influence interactions with recipient cells.

Consistent with the MISEV2023 recommendations, EVs are used throughout this review as the collective term for membrane-enclosed particles released by cells [10]. EVs are released by diverse source cells, including immune cells, mesenchymal stromal cells, tumor cells, and engineered producer cells, and differ in biogenesis, size, composition, and isolation method [17]. Their lipid bilayer can protect loaded RNA, while membrane proteins and other associated molecules may promote cellular recognition and reflect source-cell biology [18]. Figure 1 summarizes the principal EV sources, vesicle populations, and membrane- and cargo-associated features relevant to RNA delivery.

Recent EV engineering studies have addressed both therapeutic application and delivery control. EV-based formulations have been used to co-deliver antigen-encoding mRNA and protein for melanoma vaccination [19], carry *siS100A4* to the lung pre-metastatic niche after breast-cancer surgery [20], and deliver siPD-L1 to tumors and lymph nodes for immune activation and checkpoint suppression [21]. In parallel, sequence-specific CD63-PUFe recruitment has improved endogenous mRNA loading, with VSV-G incorporated to support functional delivery [22], whereas orientation-controlled RNA nanostructures have enabled either partial RNA loading or surface display of aptamer and folate ligands [23]. These approaches connect cargo incorporation with the intended cellular or anatomical site of RNA activity.

However, native EVs have notable limitations as drug delivery platforms. First, the biological properties of EVs are closely related to their RNA delivery functions. Different source cells can influence the delivery characteristics and behavior of EVs. Specifically, vesicles derived from tumor cells, immune cells, mesenchymal stromal cells, or engineered cell lines may differ in membrane proteins, endogenous cargoes, immunological activity, and interactions with recipient cells. These differences may lead to unpredictable biological effects and increase the complexity of therapeutic applications [24]. Second, native EVs have limited RNA loading efficiency and are difficult to use for efficient encapsulation of exogenous therapeutic RNA, which restricts their potential as universal RNA delivery carriers [25]. In addition, large-scale and high-purity isolation of EVs from cell culture supernatants remains technically challenging. Low yield and batch-to-batch variability make it difficult to meet the requirements for clinical translation [17].

### 2.2. Lipid-Based Nanocarriers for RNA Delivery

**Figure 2:**
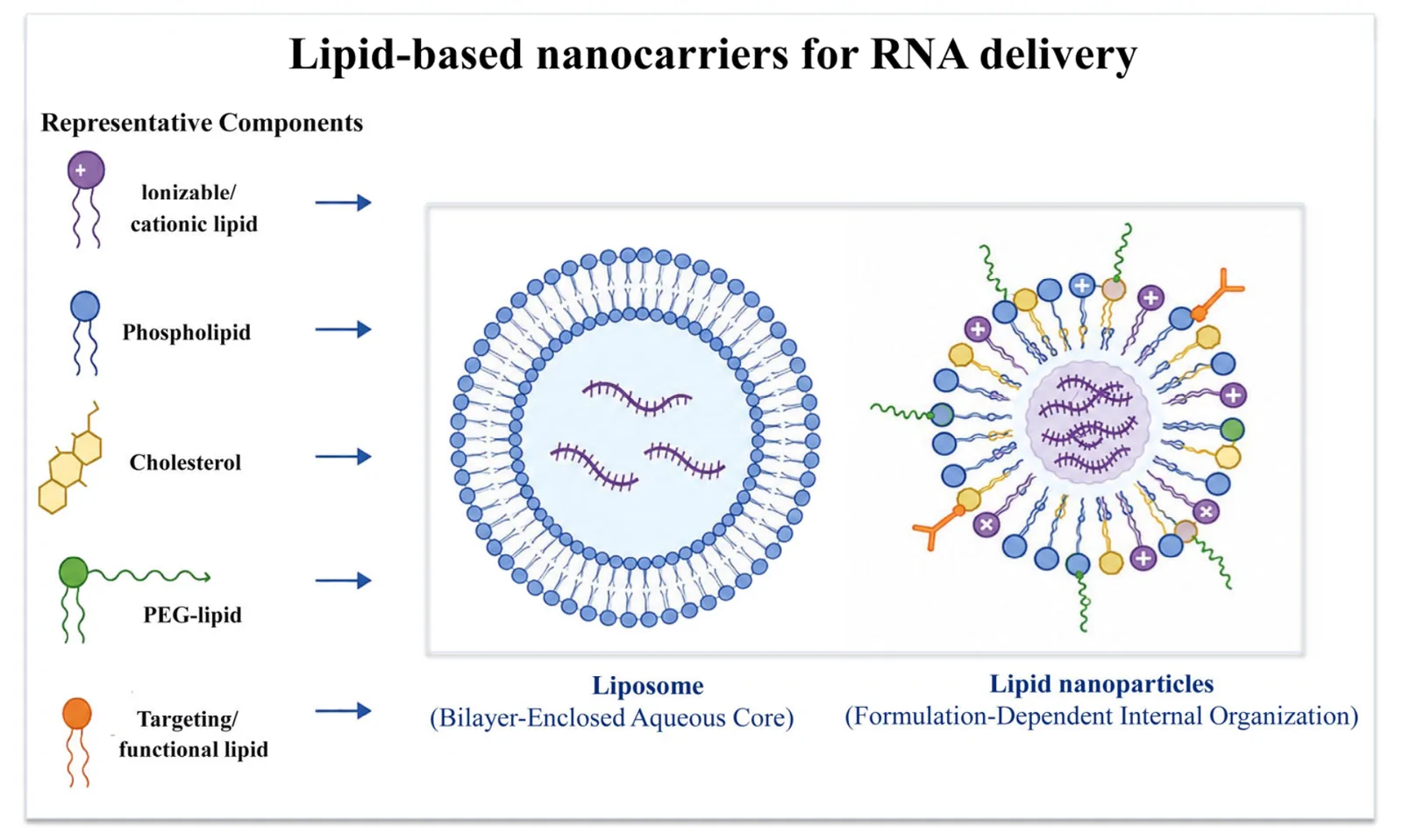
Lipid-based nanocarriers for RNA delivery. Liposomes and lipid nanoparticles (LNPs) are commonly formulated from ionizable or cationic lipids, phospholipids, cholesterol, and polyethylene glycol (PEG)-conjugated lipids, with targeting or other functional lipids incorporated when required. Liposomes contain an aqueous compartment enclosed by a lipid bilayer, whereas the internal organization of LNPs varies with their composition and formulation. The LNP structure shown here is a simplified schematic and does not represent a universal architecture.

Liposomes and lipid nanoparticles are mature synthetic lipid carriers for nucleic acid delivery. They are commonly formulated from ionizable or cationic lipids, phospholipids, cholesterol, and polyethylene glycol (PEG)-conjugated lipids, with targeting or other functional lipids incorporated when required. Liposomes typically contain an aqueous compartment enclosed by a lipid bilayer, whereas lipid nanoparticles have a more compact and formulation-dependent internal organization for RNA encapsulation [26]. Lipid composition can further influence RNA encapsulation, serum stability, biodistribution, and tolerability. Compared with cell-derived vesicles, lipid-based nanocarriers are generally easier to formulate by adjusting lipid composition, particle properties, and surface modification, thereby enabling coordinated optimization of RNA encapsulation, cellular uptake, intracellular release, and functional activity, and allowing adaptation to different types of RNA cargoes [27]. The principal lipid components and schematic organization of liposomes and lipid nanoparticles are shown in Figure 2.

Intracellular release is critical for effective drug delivery by lipid-based carriers. Ionizable lipids are commonly used because they remain relatively neutral at physiological pH but become protonated in acidic endosomal environments, thereby promoting endosomal membrane destabilization and RNA release [4]. Beyond ionizable lipids, helper lipids, cholesterol, and PEG-lipids also shape particle structure, serum stability, circulation behavior, and intracellular delivery performance [28]. The interaction between lipid nanoparticles and membrane-based biological barriers further depends on lipid composition, surface properties, and membrane dynamics, which together influence cellular entry and intracellular trafficking [7]. Environment-responsive lipid/siRNA nanoparticles illustrate how lipid chemistry can be adapted to improve cancer-related gene silencing [29]. These design principles make lipid-based carriers highly tunable, but they may also influence toxicity, immune activation, and tissue distribution. In a recent mRNA cancer-vaccine study, dendritic-cell-directed delivery was combined with a pH-responsive lipid to enhance endosomal escape and antigen expression [30].

Despite their formulation advantages, lipid-based nanocarriers face several limitations in cancer therapy [31]. Systemically administered lipid nanoparticles often show substantial liver and spleen accumulation, and efficient delivery to solid tumors or specific immune-cell subsets remains difficult [4]. Endosomal escape is also incomplete in many settings, so high cellular uptake does not always translate into strong RNA activity. Lipid nanovesicle reviews describe how lipid composition and immune context can influence cancer immunotherapy outcomes [32]. Analyses of immune responses to mRNA–lipid nanoparticle formulations further illustrate why inflammatory and tolerability signals need to be monitored during lipid-based delivery [8]. Engineered multidomain lipid nanoparticles have therefore been developed to improve cell selectivity [33]. Antibody-functionalized lipid nanocarriers provide a related route for RNA-based cancer gene therapy [34]. Lipid-based cancer immunotherapy provides additional context for therapeutic nanosystem design [35]. Repeated administration introduces an additional limitation for PEG-containing lipid nanocarriers: the accelerated blood clearance (ABC) phenomenon. An initial dose can stimulate splenic B cells to produce anti-PEG IgM; upon subsequent administration, antibody binding activates complement and promotes hepatic uptake by Kupffer cells. ABC can shorten circulation and alter biodistribution, with its magnitude influenced by PEG chemistry and surface density, lipid dose, and dosing interval [36]. These limitations have encouraged the development of modified lipid carriers, ligand-functionalized lipid nanoparticles, biomimetic lipid vesicles, and EV–lipid hybrid systems.

EVs and lipid-based nanocarriers provide complementary but distinct foundations for RNA delivery. Table 1 outlines the major components, advantages, and limitations of the two parent carrier types.

## 3. Classification and Design of EV–Lipid Hybrid Systems

The term “EV–lipid hybrid systems” is used in this review as an umbrella designation for formulations in which EVs or EV-derived membranes are intentionally combined with synthetic lipid components. This broad designation encompasses systems with different membrane arrangements and does not assume that every reported preparation consists predominantly of completely fused particles.

**Figure 3:**
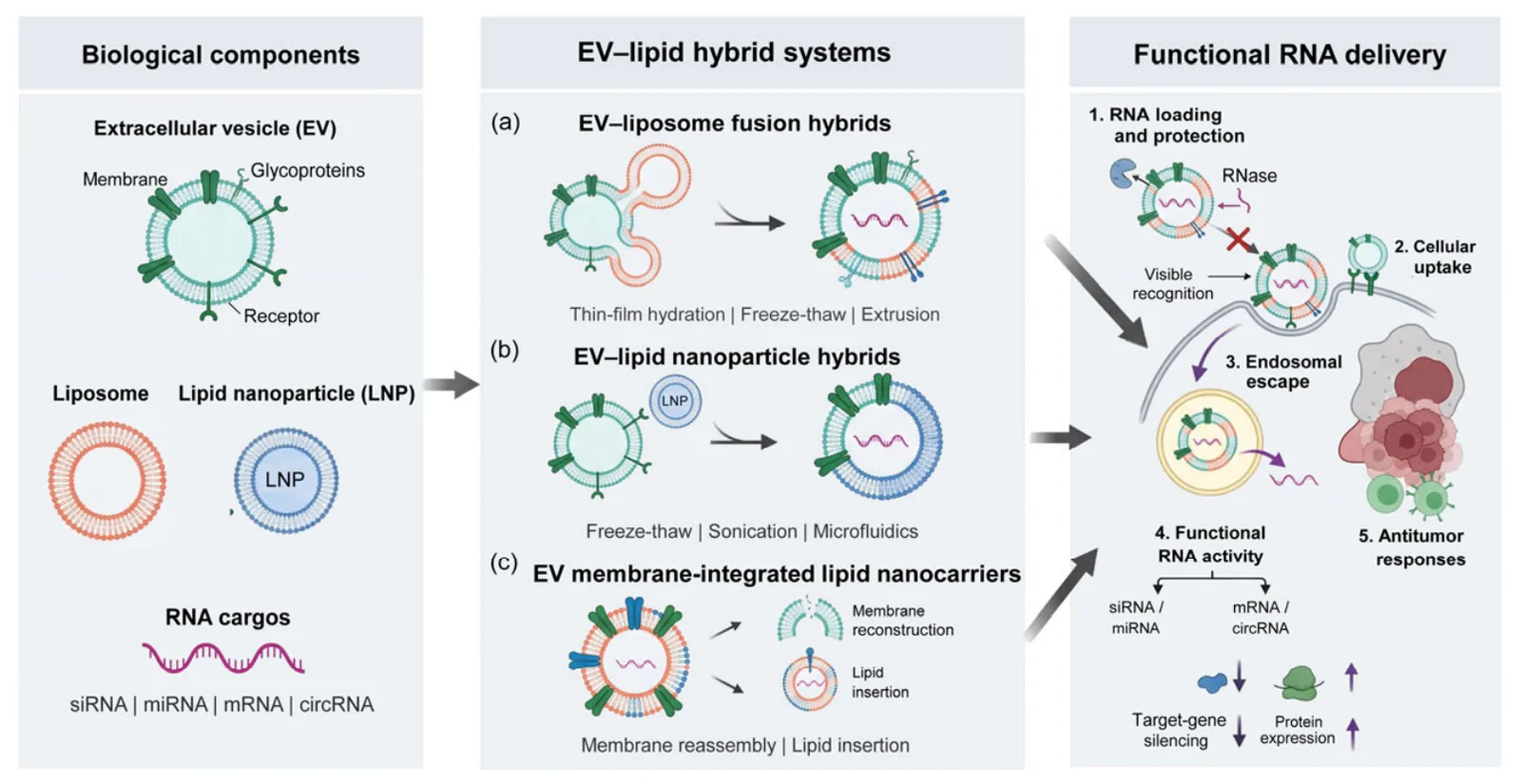
Classification and representative functional RNA-delivery processes of EV–lipid hybrid systems. The three architectures include EV–liposome fusion hybrids, EV–lipid nanoparticle hybrids, and EV membrane-integrated lipid nanocarriers. The right panel summarizes RNA loading and protection, cellular uptake, endosomal escape, functional RNA activity, and antitumor responses. The illustrated delivery pathway is schematic and may vary among formulations.

The systems are therefore organized according to both the nature of the synthetic lipid component and the manner in which it is combined with EV-derived material. Three principal categories are considered: EV–liposome fusion hybrids, EV–lipid nanoparticle hybrids, and EV membrane-integrated lipid nanocarriers. The first two categories comprise reported combinations of intact EVs with liposomes or lipid nanoparticles, whereas the third includes reassembly of isolated EV membranes with synthetic phospholipids and insertion of exogenous lipids into intact EV membranes. These structural relationships are illustrated in Figure 3. The systems included in this review are summarized in Table 2 according to their preparation method, cargo, and cancer application. Although this review focuses on RNA delivery, two plasmid-DNA studies are included because their EV–lipid hybrid architectures and preparation methods provide relevant information on carrier formulation, characterization, and cancer gene delivery. Their findings are therefore considered in relation to hybrid-system design and evaluation rather than RNA-specific delivery performance.

### 3.1. EV–Liposome Fusion Hybrids

EV–liposome fusion hybrids are produced by combining intact EVs with synthetic lipid vesicles through extrusion, sonication, freeze–thaw cycling, PEG-mediated fusion, or programmable membrane docking. The selected method can affect fusion reproducibility, particle heterogeneity, preservation of EV-associated proteins, and the amount of residual unfused EVs or liposomes. In a recent single-particle comparison, sonication and freeze–thaw cycling generated relatively high proportions of particles carrying both EV- and liposome-associated signals, whereas extrusion retained stronger EV-associated protein signals. Nevertheless, all three methods produced heterogeneous particle populations [49]. Hybrid formation and membrane-protein retention therefore need to be evaluated separately for each preparation method.

Extrusion and sonication are among the most accessible laboratory-scale approaches. Evers et al. hydrated a DLin-MC3-DMA-containing lipid film with siRNA and tumor-derived EVs before sequential extrusion, producing hybrid particles that mediated reporter-gene silencing in several cell lines [15]. Sulthana et al. similarly used extrusion to combine tumor-derived EVs with PEGylated liposomes. The resulting particles were approximately 140 nm and showed greater tumor and major-organ accumulation than the corresponding liposomes, although this study did not include a therapeutic RNA cargo [16]. Sonication combined with extrusion was subsequently used to prepare cRGD-modified hybrids carrying triptolide and surface-associated miR-497 for cisplatin-resistant ovarian cancer [38]. Related mechanical processing methods were also applied to plasmid-containing hybrid systems designed for cancer immunotherapy [43,44]. These procedures can accommodate different lipid compositions and cargo arrangements, but most of the resulting preparations were characterized by population-level measurements and did not include a purification step capable of completely removing unfused parent particles.

Freeze–thaw cycling has been used extensively for hybrids containing therapeutic RNA. Wu et al. combined *ALKBH5 *mRNA-loaded liposomes with HEK293T-derived exosomes through three freeze–thaw cycles. The resulting particles were approximately 235 nm, with a PDI of 0.24 and an mRNA encapsulation efficiency of about 89%. Delivery restored ALKBH5 expression and reduced tumor burden in both AOM/DSS and colorectal cancer patient-derived xenograft models [39]. Using a similar procedure, Gu et al. fused DR5-scFv-functionalized exosomes with cationic liposomes carrying triptolide and *CYP3A4 *siRNA. *CYP3A4 *silencing prolonged intracellular triptolide exposure, while the hybrid formulation increased its plasma half-life relative to the free drug [40]. Freeze–thaw cycling was also used to prepare M1-exosome-based photothermal hybrids carrying *IGF2BP2 *siRNA, which were approximately 204 nm and achieved an siRNA encapsulation efficiency of 89.6% [42]. Although freeze–thaw processing is operationally straightforward, repeated membrane disruption may influence the retention or orientation of EV-associated proteins, making post-processing characterization particularly important.

PEG-mediated fusion has mainly been applied to multifunctional vesicles containing both RNA and additional therapeutic components. Han et al. used PEG8000 to combine M1 macrophage-derived exosomes with cypate-containing liposomes loaded with *SerpinB9 *siRNA and granzyme B. Near-infrared irradiation promoted cargo release, and the treatment inhibited primary tumor growth; its combination with anti-PD-L1 also reduced pulmonary metastases in melanoma models [41]. PEG8000 was also used to fuse honeysuckle-derived vesicle-like nanoparticles with paclitaxel-loaded liposomes. A vesicle-to-liposome ratio of 3:1 produced particles of approximately 152 nm with a paclitaxel encapsulation efficiency of 86.6% [45]. The latter system contained endogenous plant miRNAs rather than an exogenously loaded therapeutic RNA, but it provides an additional example of PEG-mediated vesicle–liposome fusion. PEG avoids repeated mechanical processing, although polymer removal and separation of unfused particles remain necessary.

Programmable membrane docking provides greater control over individual fusion events. Malle et al. inserted complementary lipid-anchored DNA strands into EV and liposome membranes to bring mRNA-loaded liposomes into contact with EVs. A separate DNA handle enabled immobilization and removal of unfused liposomes, after which the hybrid particles were recovered through toehold-mediated strand displacement [37]. Single-particle analysis indicated predominantly one-to-one fusion and approximately two mRNA molecules per fused vesicle. The recovered particles produced mCherry expression in recipient cells, directly linking controlled fusion and purification with functional mRNA delivery. This approach offers better control over particle formation than physical processing methods but requires additional synthetic components and purification steps.

Overall, extrusion, sonication, freeze–thaw cycling, and PEG-mediated fusion provide relatively straightforward routes for preparing EV–liposome hybrids with diverse RNA cargoes and treatment components, whereas programmable docking offers greater control over fusion and purification. Direct comparison of delivery performance remains difficult because the studies differ in EV source, lipid composition, cargo location, purification procedure, tumor model, and outcome measurement. These differences are summarized in Table 2.

### 3.2. EV–Lipid Nanoparticle Hybrids

EV–lipid nanoparticle hybrids retain intact EVs while introducing lipid nanoparticle formulations developed specifically for nucleic-acid loading. Their synthetic phase more often contains ionizable lipids and is produced by ethanol injection or microfluidic mixing.

The two reported EV–lipid nanoparticle systems were developed with different priorities. Abdel-Bar et al. [14] focused on formulation optimization by combining siRNA-loaded DLin-MC3/DOPE/cholesterol/C16-PEG2000 ceramide nanoparticles with exosomes derived from five cancer cell types. Freeze–thaw cycling and sonication were evaluated using a Box–Behnken design to determine how processing conditions affected particle size and fusion efficiency. An exosome-to-lipid nanoparticle ratio of 2:1 produced particles smaller than 170 nm with fusion efficiencies above 50%, while the EV-associated proteins CD9, CD63, and CD81 remained detectable. In contrast, Hu et al. [46] incorporated hybridization into a therapeutic co-delivery strategy for pancreatic ductal adenocarcinoma. IL-12 circRNA and gemcitabine were co-assembled with ALC-0315, cholesterol, DSPE-PEG2000-maleimide, and DPPC by microfluidic mixing. The resulting lipid nanoparticles were combined with Pan02-derived exosomes and extruded 21 times through a 200 nm membrane, yielding hybrid particles of approximately 126 nm that remained stable for 14 days under the reported storage conditions.

EV–lipid nanoparticle hybrids have been examined in fewer studies than EV–liposome fusion systems. Even so, the two available studies provide examples of siRNA and circRNA delivery.

### 3.3. EV Membrane-Integrated Lipid Nanocarriers

EV membrane-integrated lipid nanocarriers do not require the fusion of two intact vesicles. Instead, synthetic lipids are used either to reconstruct isolated EV membranes or to modify the surface of intact EVs. This approach makes membrane composition a direct design variable and may reduce unwanted luminal EV cargo. It also raises a different set of questions, particularly concerning membrane orientation, protein retention, inserted-lipid stability, and the fate of residual endogenous material.

Membrane reconstruction was used by Zhou et al. [47] to deliver *siRNA targeting cyclin-dependent kinase 1 (CDK1)* in hepatocellular carcinoma. Tumor-derived EVs were disrupted, and their membrane components were reassembled with DPPC by thin-film hydration and extrusion. FRET analysis guided selection of the EV-membrane-to-phospholipid ratio. The *CDK1-targeting siRNA* was then loaded by electroporation at 400 mV and 125 µF, followed by Sephadex G-50 purification. The resulting nanovesicles measured approximately 117 nm. They retained tumor-EV membrane proteins while excluding much of the original luminal cargo.

A less disruptive option is post-isolation lipid insertion. Zhan et al. [48] incubated reticulocyte-derived exosomes with L-α-phosphatidylcholine at 37 °C for 30 min, followed by purification by ultracentrifugation. The modification caused little reported change in particle size, morphology, marker profile, or loading capacity. Cholesterol-conjugated anti-miR-21 was subsequently loaded into the engineered exosomes.

These studies represent two different levels of membrane engineering. Reassembly offers broader control over carrier composition, whereas lipid insertion preserves the intact EV and requires fewer processing steps. Both differ fundamentally from whole-vesicle fusion because the membrane itself becomes the principal object of design. Their potential advantage is structural control. How consistently native membrane topology and function survive either process remains less certain.

### 3.4. Verification of Hybrid Formation and Particle Purity

The evidence used to identify EV–lipid hybrid structures is not uniform across the literature, and the reported assays often address different aspects of particle composition. Dynamic light scattering, nanoparticle tracking analysis, and conventional transmission electron microscopy provide information on particle size, concentration, and morphology, but these measurements are not specific to membrane fusion. Similarly, the detection of EV-associated proteins by Western blotting confirms that these components remain within the preparation, although it does not establish their incorporation into the same membrane when residual EVs have not been removed.

FRET assays are among the most frequently used approaches for examining interactions between EVs and synthetic lipid membranes. Abdel-Bar et al. quantified fluorescence dequenching after freeze–thaw or sonication processing and used the resulting signal to compare formulation conditions [14]. Zhou et al. applied FRET to select the ratio between isolated tumor-EV membranes and DPPC during membrane reassembly [47]. In these settings, changes in FRET provide useful information on the redistribution of labeled lipids within the particle population. However, similar changes may also accompany lipid exchange, hemifusion, or fusion involving only a fraction of the particles. FRET is therefore most appropriately interpreted as evidence of population-level lipid mixing rather than direct confirmation that the entire preparation consists of fully fused vesicles. Dual-fluorescence colocalization addresses a related but distinct aspect of hybrid formation by showing that EV-derived and lipid-derived signals remain associated. Close membrane apposition, aggregation, or surface adsorption may nevertheless produce overlapping signals, particularly in positively charged formulations in which electrostatic interactions contribute to particle association, as in the cationic liposome-based system reported by Tong et al. [43].

Clearer structural assignments have been obtained when membrane or content mixing was examined together with suitable controls and post-fusion purification. Malle et al. combined a content-mixing assay with sequence-specific membrane docking, non-complementary DNA controls, and real-time single-particle imaging. Unfused liposomes were removed before the resulting particles were recovered by toehold-mediated release, allowing individual fusion events to be distinguished from transient membrane contact [37]. Evers et al. used a complementary approach in which particles were captured through the EV-associated tetraspanins CD9, CD63, or CD81. Detection of both a synthetic phospholipid and fluorescent siRNA after washing supported the presence of EV-associated and synthetic components within the captured fraction. The authors nevertheless acknowledged that intact EVs could not be completely excluded from the final preparation [15]. Han et al. further combined Nycodenz density-gradient purification with a physical-mixture control, fluorescence colocalization, flow cytometry, FRET, and EV-marker analysis, providing comparatively well-supported evidence of membrane integration after PEG-mediated processing [41]. Beyond fluorescence-based measurements, cryogenic electron microscopy (cryo-EM) has been used to visualize membrane contact and fusion intermediates between EVs [50]. Although that study did not examine EV–lipid hybrid particles, it illustrates how cryogenic imaging can provide ultrastructural information on membrane remodeling when applied to purified hybrid preparations.

Other formulations have been characterized primarily through particle morphology, size, surface charge, or retention of EV-associated proteins. For example, the *ALKBH5 mRNA formulation* described by Wu et al. displayed a spherical morphology and defined physicochemical properties after freeze–thaw processing, but formation-specific analysis beyond these measurements was not reported [39]. Similarly, the Pan02 exosome–LNP formulation reported by Hu et al. was supported by particle morphology, size, and the retention of exosomal markers after extrusion, without a fusion-specific assay capable of distinguishing hybrid particles from residual parent vesicles [46]. These systems differ from the membrane-reassembled nanovesicles reported by Zhou et al., in which isolated EV membrane components were combined with phospholipids [47]. Phosphatidylcholine-engineered exosomes represent another distinct configuration because exogenous lipid molecules were inserted into otherwise intact EV membranes rather than introduced through whole-vesicle fusion [48].

The interpretation of hybrid formation is also closely linked to the purification procedure. Size-exclusion chromatography and ultrafiltration are useful for removing free RNA and small molecular components, but EVs, liposomes, and hybrid particles may have overlapping size distributions and may therefore remain in the same collected fraction. Asymmetric-flow field-flow fractionation (AF4) has been used to resolve exomeres and small-EV subpopulations according to their hydrodynamic properties [51]. Its application to EV–lipid hybrid preparations would require experimental confirmation that residual EVs, lipid particles, and hybrid particles can be resolved under the selected separation conditions.

Density-gradient separation, affinity-based enrichment, or programmable capture-and-release strategies may provide greater resolution when differences between the parent and hybrid populations can be demonstrated experimentally. Proteomic or lipidomic profiling and topology-sensitive assays can add information on the retention and orientation of EV-associated membrane components, although these analyses are most informative when performed on a purified particle fraction and interpreted alongside structural measurements [10]. The most reliable assignment of hybrid identity consequently comes from the convergence of post-processing purification, complementary structural or compositional analyses, and comparisons with EV-only, lipid-only, and unfused physical-mixture controls.

## 4. RNA Loading, Cellular Uptake, and Functional RNA Delivery

The structural categories described in Section 3 explain how EVs and synthetic lipids are combined, but carrier structure alone does not ensure successful RNA delivery. RNA must remain associated with the hybrid particle and resist extracellular degradation. It must then enter the target cell, reach the cytosol or another required intracellular site, and retain its biological activity [27,52]. Failure at any step can reduce delivery efficiency even when cellular uptake is high [53,54]. Accordingly, this section focuses on RNA loading and protection, cellular uptake, intracellular trafficking, cytosolic RNA release, and the assays used to confirm functional RNA delivery.

### 4.1. RNA Loading and Protection

In most EV–lipid hybrid systems, the synthetic lipid component provides the main site for RNA loading, although the time at which RNA is introduced varies among formulations. In several studies, siRNA, mRNA, or circRNA was first associated with a liposome or lipid nanoparticle, followed by fusion with EVs [14,39,40,41,46]. This approach allows the lipid composition, surface charge, and RNA-to-lipid ratio to be adjusted before the biological membrane is introduced [27]. RNA loading can therefore be optimized separately from EV–lipid fusion. However, efficient loading into the parent lipid carrier does not necessarily mean that RNA remains protected after freeze–thaw cycling, sonication, extrusion, or PEG-mediated fusion.

Other studies introduced RNA during or after hybrid-particle formation. Evers et al. incorporated siRNA during lipid-film hydration and extrusion in the presence of EVs [15]. In the miR497/triptolide system, the drug was retained in the lipid compartment, whereas calcium-phosphate-associated miR497 was assembled at the particle surface [38]. Similarly, the positive surface charge of a photothermal hybrid was used to complex *IGF2BP2-siRNA* after fusion [42]. By contrast, *siCDK1* was loaded into reconstructed EV-membrane lipid vesicles by electroporation [47], while cholesterol-conjugated anti-miR-21 was introduced after phosphatidylcholine insertion into intact exosomes [48]. Thus, RNA may be encapsulated within the lipid phase, incorporated during membrane reconstruction, or associated with the outer surface of the final particle.

Malle et al. integrated programmable fusion with immobilization and toehold-mediated release, allowing unfused liposomes to be removed before mRNA-loaded hybrid particles were collected [37]. By comparison, most physical fusion methods do not include a similar purification step. Their apparent encapsulation values may therefore include free RNA, RNA associated with residual parent particles, or cargo carried by incompletely fused vesicles.

Different loading methods also require different forms of characterization. For preloaded lipid carriers, RNA encapsulation and nuclease protection should be confirmed after fusion. Surface-complexed RNA should remain stably associated with the carrier but still be released after cellular entry. After electroporation, free RNA should be removed, and the amount retained by the vesicles should be measured. In all cases, total RNA or fluorescence alone may include free cargo and unfused parent particles. Purification, nuclease-protection assays, and appropriate parent-particle controls are therefore needed [10]. Because the reported studies differ in RNA location, purification procedure, and the methods used to calculate loading or encapsulation efficiency, the available data do not support ranking one loading route as the most efficient. A valid comparison would require loading, RNA integrity, and functional activity to be measured after fusion and purification using matched parent carriers.

### 4.2. Cellular Uptake, Intracellular Trafficking, and Cytosolic RNA Release

Once RNA has been retained by the carrier, cellular entry becomes the next barrier. EV-derived membrane components may influence cell binding and uptake in a source-dependent manner, whereas synthetic lipids and added ligands influence charge, protein adsorption, and receptor engagement [9,52]. Abdel-Bar et al. showed that the association of optimized exosome–lipid nanoparticle hybrids with cancer cells depended partly on the source of the exosomes [14]. Other systems introduced cRGD, folate, or DR5-scFv-binding fragments with the aim of promoting receptor-mediated cellular interactions [38,39,40]. M1 macrophage membranes were instead used in photothermal hybrids intended to interact with tumors and inflammatory compartments [41,42]. When an EV membrane and an external ligand are present together, increased uptake cannot be assigned to the EV component without matched controls that isolate each surface contribution.

Among the hybrid systems reviewed, the most detailed analysis of post-entry trafficking was performed with tumor-derived EV membrane–phospholipid hybrid nanovesicles (LEVs). In Sk-hep1 cells, inhibitor studies implicated clathrin- and caveolae-dependent endocytosis and macropinocytosis. At 4 h, DiI-labeled LEVs showed less colocalization with LAMP1-positive lysosomes and greater colocalization with Golgin-97-positive Golgi (approximately 40%) and KDEL-positive endoplasmic reticulum (approximately 60%) than the liposome control; a similar distribution was reported in tumor sections [47]. The authors interpreted this pattern as endosome-to-Golgi-to-ER trafficking that reduced lysosomal degradation. Because the experiments followed the labeled nanovesicle membrane rather than *siCDK1 itself*, they establish altered intracellular sorting but not the release of siRNA across the endosomal membrane into the cytosol.

A complementary observation came from phosphatidylcholine-engineered exosomes [48]. Lipid insertion increased uptake in glioblastoma and breast-cancer cells without a substantial reported change in vesicle size or marker profile. The finding suggests that membrane lipid composition alone can alter cell interaction. It does not, however, identify a single receptor or internalization pathway.

The other four hybrid studies provided functional, rather than direct imaging, support for cytosolic RNA availability. Evers et al. found that EV–liposome hybrids produced luciferase silencing in three reporter cell lines; in U87-MG cells, knockdown remained comparable to liposomes despite lower particle uptake, which the authors presented as a possible indication of more efficient cytosolic siRNA delivery [15]. Abdel-Bar et al. quantified Atto740-siRNA association and target-protein knockdown after treatment with exosome–LNP hybrids, but did not examine endosomal localization or cytosolic siRNA release [14]. In the programmable EV–liposome study, expression of mCherry protein confirmed that the delivered mRNA remained available for translation, although the intracellular path of the mRNA was not tracked [37]. Likewise, *ALKBH5 mRNA* and protein restoration after treatment with ALKBH5-loaded exosome–liposome hybrids demonstrated functional mRNA delivery without identifying the route of endosomal escape [39].

These distinctions are important because gene silencing or protein expression requires cytosolic access but does not quantify how much RNA was released, when release occurred, or which compartment was crossed. Image-based studies of LNP formulations have estimated that only a small fraction of internalized siRNA reaches the cytosol and have visualized the limited time window of release [55,56]. Outside the hybrid/fusion category, cholesterol-enriched exosomes have been reported to deliver siRNA through direct plasma-membrane fusion, providing a mechanistically distinct EV-based comparator [57].

Endosomal escape remains quantitatively inefficient because particle uptake, endosomal membrane damage, and cytosolic RNA release are distinct events. Galectin-9 imaging can quantify endosomal membrane damage separately from particle uptake and protein expression [58]. Single-molecule localization microscopy has resolved individual LNP–mRNA particles and rare mRNA-release events from endosomal recycling tubules [59]. Live-cell and super-resolution microscopy further showed that only a subset of internalized LNPs induced galectin-positive membrane damage, that only a small fraction of endosomal RNA reached the cytosol, and that ESCRT-associated membrane perturbation was not necessarily accompanied by productive RNA release [60]. Although these studies examined conventional LNPs, their methods provide relevant tools for directly evaluating endosomal escape and cytosolic RNA release in EV–lipid hybrid systems.

### 4.3. Functional Activity of Delivered RNA

Functional RNA delivery should be evaluated according to the type of RNA cargo. For siRNA, functional delivery is demonstrated by a sequence-specific reduction in the target transcript or protein. Reporter knockdown demonstrated functional siRNA delivery in an early EV–liposome formulation [15]. Later studies examined cancer-related targets, including CD24, CD44, CD47, CYP3A4, CDK1, *SerpinB9*, and *IGF2BP2* [14,40,41,42,47]. These studies connected RNA delivery with a molecular change. Changes in phagocytosis, drug metabolism, granzyme B sensitivity, or ferroptosis are more convincing when target-gene silencing is also confirmed.

For mRNA and circRNA, successful delivery should result in detectable protein expression. Programmable mRNA transfer was confirmed by mCherry expression after delivery of fused EV–liposome particles [37]. In a therapeutic system, *ALKBH5 mRNA* restored ALKBH5 protein and regulated the JMJD8/PKM2 metabolic pathway [39]. IL-12 circRNA delivery was evaluated through cytokine expression and subsequent immune activation [46]. Reporter expression shows that mRNA remains translatable after delivery, while restoration of a therapeutic protein further connects delivery with the intended pathway.

For miRNA and anti-miRNA delivery, changes in the intended regulatory pathway should also be examined. The miR497 and anti-miR-21 systems were evaluated through changes in related signaling pathways, target proteins, and cellular responses [38,48]. Because one miRNA can regulate several transcripts, these pathway-level effects are less specific than direct siRNA knockdown. Appropriate controls are particularly important when miRNA delivery is combined with other active components.

The hybrid literature currently supports three different levels of intracellular assessment: particle association, organelle-level trafficking, and cargo-specific functional activity. Zhou et al. examined post-endocytic organelle distribution, whereas the other hybrid studies inferred cytosolic availability from knockdown or protein expression. Among the five studies examined above, none quantified the cytosolic fraction of released RNA or directly visualized RNA crossing the endosomal membrane. Direct release measurements used together with carrier tracking and cargo-specific activity would connect intracellular localization with functional RNA delivery.

The principal measurements and controls needed to distinguish hybrid formation, cellular uptake, and functional RNA delivery are summarized in Table 3.

## 5. Cancer-Specific Applications of EV–Lipid Hybrid RNA Delivery

EV–lipid hybrid systems have been used for RNA delivery in several tumor models. Their current applications include gastrointestinal, hepatobiliary, gynecologic, skin, head and neck, and brain tumors. In these studies, RNA has been delivered alone or together with chemotherapy, photothermal treatment, and immunotherapy.

### 5.1. Gastrointestinal and Hepatobiliary Cancers

Colorectal cancer, pancreatic ductal adenocarcinoma, and hepatocellular carcinoma are biologically distinct malignancies. Molecular heterogeneity affects treatment selection in colorectal cancer, whereas the dense stroma of pancreatic cancer restricts perfusion and drug penetration. Hepatocellular carcinoma is further complicated by the underlying chronic liver disease in many patients [61,62,63].

Across gastrointestinal and hepatobiliary cancers, EV–lipid hybrids have served three distinct therapeutic roles. In colorectal cancer, *ALKBH5 mRNA* replacement restored ALKBH5 expression, reduced JMJD8/PKM2-associated glycolysis, and suppressed tumor growth in AOM/DSS and patient-derived xenograft models [39]. In pancreatic ductal adenocarcinoma, IL-12 circRNA was combined with gemcitabine after losartan pretreatment; the resulting antitumor activity reflected improved tumor perfusion, cytokine expression, and chemotherapy [46]. In hepatocellular carcinoma, *siCDK1* acted as the principal therapeutic cargo, reducing intratumoral *CDK1* mRNA expression and inhibiting tumor growth in c-Myc-overexpressing tumors [47]. These studies therefore illustrate protein replacement, RNA-supported combination therapy, and direct gene silencing, respectively.

### 5.2. Breast and Ovarian Cancers

Breast cancer consists of several molecular subtypes, and treatment is selected according to receptor status and tumor biology. Platinum-based chemotherapy remains important in epithelial ovarian cancer, but acquired resistance is frequent after repeated treatment [64,65].

The breast- and ovarian-cancer formulations addressed different mechanisms of tumor persistence. In the 4T1 breast cancer model, simultaneous silencing of CD24, CD44, and CD47 increased macrophage-mediated tumor-cell clearance and delayed tumor growth, thereby addressing stem-like properties and immune escape [14]. In cisplatin-resistant ovarian cancer, miR-497 was combined with triptolide to suppress PI3K/AKT/mTOR signaling, increase reactive oxygen species accumulation, and promote apoptosis [38]. Thus, one system silenced surface molecules associated with resistance to immune clearance, whereas the other was designed to reverse a defined mechanism of chemotherapy resistance.

### 5.3. Melanoma and Head and Neck Cancers

Melanoma has a high metastatic potential and can rapidly acquire resistance to systemic treatment. Head and neck squamous cell carcinoma also displays marked molecular heterogeneity and is commonly treated with multimodal regimens [66,67]. In these tumors, RNA has mainly been combined with a second therapeutic modality.

In melanoma and head and neck squamous cell carcinoma, siRNA was used primarily to increase tumor sensitivity to an accompanying treatment. *CYP3A4* silencing prolonged triptolide exposure in metastatic melanoma, while the DR5-scFv-directed component supported tumor recognition and apoptotic signaling [40]. *SerpinB9* silencing instead increased susceptibility to granzyme B and was combined with photothermal treatment and anti-PD-L1 therapy [41]. In head and neck squamous cell carcinoma, *IGF2BP2* knockdown weakened NRF2-dependent antioxidant defenses and enhanced ferroptosis induced by mild photothermal treatment [42]. Although these systems acted through drug metabolism, granzyme resistance, and antioxidant signaling, respectively, they shared a common therapeutic strategy in which RNA interference removed a specific resistance mechanism and enhanced the activity of a second treatment.

In addition to these RNA systems, two plasmid-DNA studies, both of which included melanoma models, expanded the use of EV–lipid hybrids for gene immunotherapy. One encoded a PD-L1 trap, whereas the other delivered an IL-12 plasmid in combination with radiotherapy [43,44]. These studies provide information on hybrid construction and cancer immunotherapy but are not included as evidence of RNA delivery.

### 5.4. Brain Tumors

Glioblastoma is highly infiltrative and remains difficult to treat after surgery, radiotherapy, and temozolomide. The blood–brain barrier and immunosuppressive tumor microenvironment further restrict drug delivery and therapeutic response [68].

Zhan et al. used phosphatidylcholine-engineered exosomes to deliver cholesterol-conjugated anti-miR-21 to U87 cells [48]. The treatment reduced miR-21 activity and enhanced the in vitro antitumor effect. However, no orthotopic brain-tumor model was included. This study therefore supports cellular anti-miR-21 delivery, but does not yet demonstrate effective RNA transport across the blood–brain barrier in vivo. More recently, Liu et al. administered a paclitaxel-loaded honeysuckle vesicle-liposome hybrid through the intranasal route [45]. The formulation improved brain and glioma distribution and prolonged survival in C6 glioma-bearing mice. Functional plant miRNAs, including miR2911, were detected in the source vesicles and were associated with macrophage polarization. The latter finding represents intranasal nose-to-brain delivery and should not be interpreted as evidence of systemic passage across an intact blood–brain barrier.

At present, the application of EV–lipid hybrid RNA delivery to brain tumors remains at an early stage. Further studies should confirm RNA accumulation and activity in orthotopic tumors.

## 6. Safety Considerations and Clinical Translation

### 6.1. Safety Considerations

Safety evaluation of the reported EV–lipid hybrids has focused mainly on short-term tolerability. Most studies monitored body weight, hematological or serum biochemical parameters, and histological changes in major organs [38,39,40,41,42,46,47]. Hemolysis was examined in the pancreatic and head and neck cancer studies [42,46], whereas the tumor-derived EV membrane formulation was additionally assessed for inflammatory cytokines and retention of the growth-promoting activity of the source EVs [47]. Transcriptomic analysis was used to examine possible off-target effects of *IGF2BP2-*siRNA [42]. No marked systemic injury was reported at the tested doses, but differences in dose, administration route, treatment duration, and safety endpoints prevent direct comparison among formulations. The available findings therefore primarily describe acute or short-term tolerability.

Evidence from non-hybrid EV studies indicates that immune responses may depend on both vesicle origin and repeated exposure. Although EVs are often regarded as weakly immunogenic [69], repeated intravenous administration of human cell-derived EVs in macaques was accompanied by EV-reactive immunoglobulin G and reduced circulation time [70]. Allogeneic exosomes also induced a T-cell response in mice under inflammatory conditions [71]. These findings do not establish that EV–lipid hybrids produce the same responses, but they show that source-dependent immunogenicity cannot be inferred from single-dose tolerability alone and support repeated-dose assessment of antibodies and cellular immune responses.

The biological properties of the source cells should also be considered, especially for tumor-derived EVs, which may carry oncogenic molecules or procoagulant membrane proteins. Removing the luminal contents, as performed in the preparation of tumor-derived EV membrane hybrid nanovesicles, may reduce this concern [47]. Tissue factor-positive EVs released by cancer cells can activate coagulation and promote thrombosis in experimental models [72]. Evaluation of tumor-derived hybrids should therefore extend beyond routine organ histology to include residual cellular components and, where appropriate, coagulation-related measurements.

The lipid component introduces a different set of concerns. Permanently cationic lipids facilitate RNA association and cellular entry, but positively charged lipid nanoparticles have been associated with liver injury and inflammatory cytokine production [73]. Positively charged formulations may also interact with fibrinogen and platelets and cause coagulation abnormalities under some conditions [74]. These findings do not mean that every cationic formulation will produce the same response, since toxicity is influenced by lipid composition, dose, particle size, and route of administration. Ionizable lipids that are largely neutral at physiological pH and become protonated in the acidic endosomal environment may provide a better balance between RNA delivery and systemic tolerability. PEG-containing lipids also warrant attention because anti-PEG antibodies, accelerated clearance, and complement-related infusion reactions have been reported for PEGylated liposomes [36].

### 6.2. EV Heterogeneity, Manufacturing, and Clinical Translation

EV heterogeneity has practical consequences for both hybrid formation and dose definition. EV yield and molecular composition may change with producer cell, passage number, cell density, activation state, and harvest conditions. Only a subset of vesicles may fuse efficiently with a given lipid carrier, so bulk measurements of size, FRET, or RNA loading can conceal residual EVs, residual lipid particles, and hybrid subpopulations with different membrane-protein content [49]. The EV source, particle number, EV-associated protein, lipid amount, and RNA dose should therefore be reported together when possible, and source- and process-matched batches should be compared in parallel.

For GMP-compatible production, qualified producer-cell banks and raw materials should be combined with defined culture conditions, documented in-process controls, and aseptic processing. Scalable workflows may use tangential-flow filtration for concentration and buffer exchange, followed by size-exclusion chromatography or another validated method for purification. The process should remove free RNA, excess lipids, residual fusion reagents, and unfused parent particles while preserving RNA integrity and biological activity. Process validation and batch-comparability assessment will also be required during scale-up.

Release testing should cover the identity of the intended hybrid architecture, particle concentration and size distribution, RNA integrity and content, relevant EV-associated markers, residual parent particles and process-related impurities, sterility, endotoxin, storage stability, and cargo-specific potency. MISEV2023 provides recommendations for EV separation and characterization [10], whereas the quality and safety framework of Takakura et al. and the ISEV and EVOLVE position papers address manufacturing, quality control, and clinical development of therapeutic EV products [75,76,77]. Because EV–lipid hybrids contain both biological membranes and synthetic lipids, specifications should be established for the purified hybrid formulation rather than extrapolated directly from either parent system.

Clinical experience with therapeutic EVs provides an indirect reference point. A phase I study evaluated allogeneic bone marrow mesenchymal stromal cell-derived exosomes carrying *KRAS^G12D*-specific siRNA in patients with metastatic pancreatic cancer [78]. Repeated intravenous administration was well tolerated at the doses examined, with no treatment-related adverse events and no maximum tolerated dose identified. Although this study did not evaluate an EV–lipid hybrid, it supports the feasibility of repeated administration of an EV-based siRNA product. The synthetic lipid components and hybrid membrane architecture will nevertheless require separate evaluation of immune responses, blood compatibility, and product consistency.

**Figure 4:**
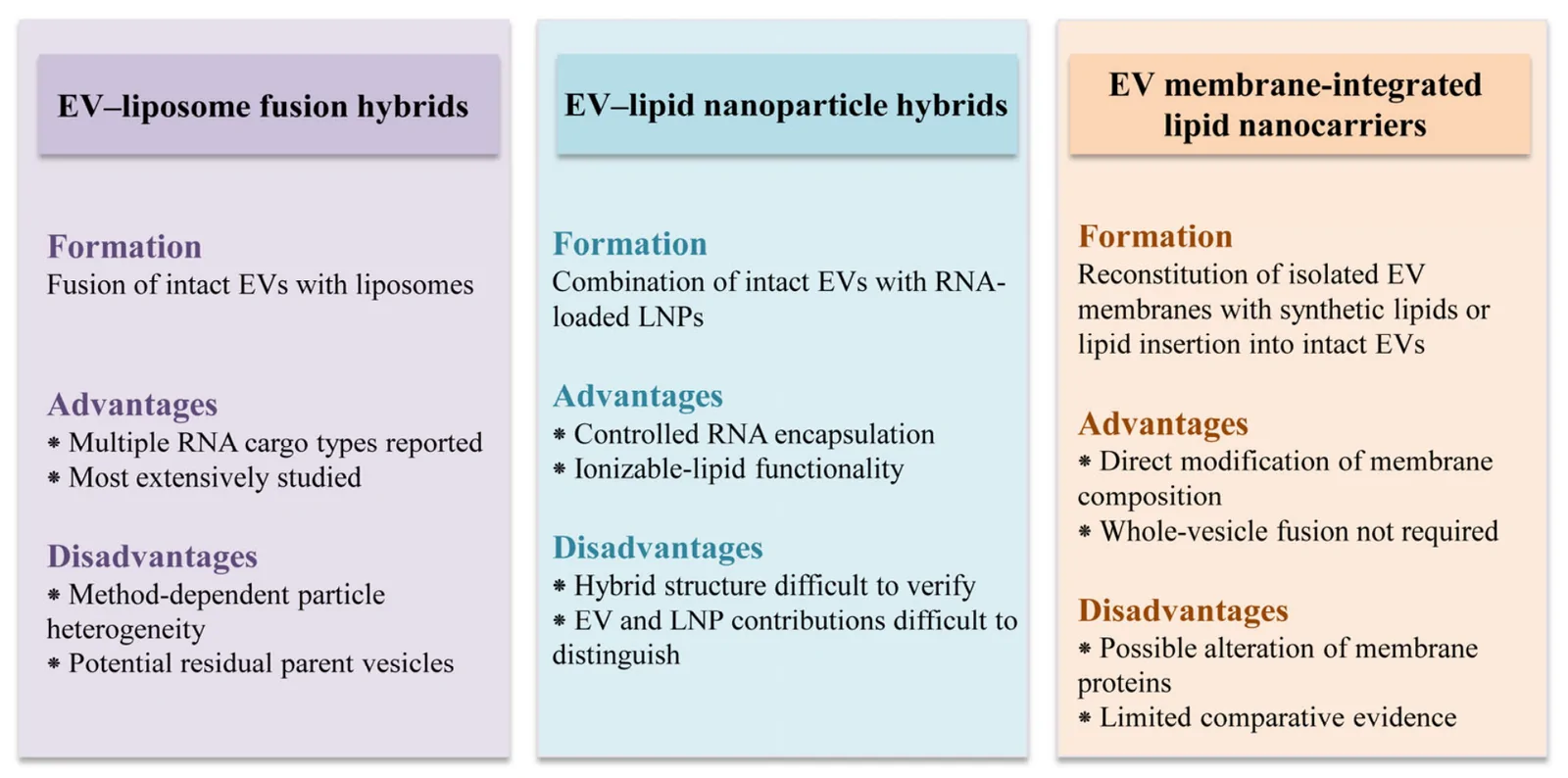
Summary of the formation, principal advantages, and disadvantages of the three major EV–lipid hybrid systems.

The principal advantages and disadvantages of the three major EV–lipid hybrid systems are summarized in Figure 4.

## 7. Conclusions

EV–lipid hybrid systems combine the biological membrane properties of EVs with the formulation flexibility of liposomes and lipid nanoparticles. Current studies show that these systems can carry different forms of therapeutic RNA and can be adapted for gene silencing, protein expression, and combination treatment in several tumor models. Their performance, however, is influenced by the EV source, lipid composition, preparation method, and RNA-loading strategy.

EV–liposome fusion has been investigated across the broadest range of RNA cargoes, preparation methods, and tumor models, although direct comparisons have not established its superiority over other hybrid formats. Important unresolved issues include heterogeneity and residual parent particles in hybrid preparations, preservation of EV-associated membrane components and functions after processing, limited quantitative evidence of cytosolic RNA release, and insufficient data on batch reproducibility, biodistribution, and safety after repeated administration. Further development will require scalable purification, complementary single-particle and ultrastructural characterization, matched EV-only, lipid-only, and physical-mixture controls, direct measurement of cytosolic RNA delivery, cargo-specific functional assessment, and systematic in vivo safety studies. Such evidence will be needed to define the hybrid architectures best suited to particular RNA cargoes, tumor types and therapeutic applications.

## Figures and Tables

**Table 1:** Extracellular vesicles and lipid-based nanocarriers in RNA delivery.

Carrier Type	Composition and RNA-Loading Control/Efficiency	Reproducibility and Scalability	Manufacturing Complexity	Clinical Maturity	Principal Strengths and Constraints
Extracellular vesicles	Cell-derived membranes with source-dependent proteins, lipids, and endogenous cargo; exogenous loading is variable and method-dependent	Sensitive to cell source, culture, isolation, and storage; scale-up remains process-dependent	Requires producer-cell expansion, conditioned-medium processing, EV isolation and purification, and source-specific quality control.	Early clinical evaluation for selected EV products	Biological membrane interface and cargo protection; variable identity, purity, loading control, and batch consistency
Lipid-based nanocarriers	Defined synthetic lipids; loading can be controlled through lipid/RNA ratio and mixing conditions	Established formulation and scale-up methods, particularly for LNPs	Requires controlled lipid mixing, solvent removal or buffer exchange, purification, sterile processing, and physicochemical quality control.	Multiple approved nucleic-acid products	Tunable composition and efficient RNA loading; liver/spleen exposure, incomplete escape, and inflammatory or repeat-dose effects

**Table 2:** Structural architectures, formulation attributes, and biological performance of EV–lipid hybrid systems relevant to cancer-related nucleic acid delivery.

Hybrid Architecture	Hybrid System	Preparation Strategy and Process Complexity	Cargo and Loading/Encapsulation	Size/PDI	Circulation, Biodistribution, and Cellular Uptake	Cancer Model and Principal Biological Outcome	References
EV–liposome fusion hybrids	EV–liposome hybrid nanoparticles	Thin-film hydration with siRNA, followed by sequential extrusion in the presence of EVs (multistep)	Firefly-luciferase-targeting siRNA; siRNA EE decreased with increasing EV content and was approximately 50% for the 1:50 EV-protein-to-lipid hybrid	~150 nm by DLS; ~100 nm by NTA; PDI ~0.2	In vivo circulation and biodistribution NR	Dose-dependent luciferase silencing in vitro; lower than liposomes in SKOV3 and HEK293T cells and comparable in U87-MG cells; no in vivo study	[15]
EV–liposome fusion hybrids	4T1 tumor-derived EV–liposome hybrids (LEVs)	Sonication followed by membrane extrusion (two post-isolation operations)	Fluorescent-liposome tracing; no therapeutic RNA loaded	140 ± 5 nm; PDI 0.145 ± 0.02	Both formulations reached tumors at 1 h, but LEVs showed more distinct tumor localization at 3–24 h and greater tumor and major-organ retention than liposomes; carrier half-life NR.	Orthotopic breast cancer and lung-metastasis localization study; no RNA-specific or therapeutic efficacy endpoint	[16]
EV–liposome fusion hybrids	DNA-programmable liposome–EV fusion particles (EVLs)	LiNA-mediated docking, bead immobilization, washing, and toehold-mediated release (highly controlled multistep process)	*mCherry mRNA*; 0.0011 ± 0.0001 fg mRNA/particle (~2 copies/particle)	141.5 ± 2.9 nm; PDI NR	In vivo circulation and biodistribution NR	mCherry expression in HEK293-H cells after delivery of 33 ng mRNA; no in vivo study	[37]
EV–liposome fusion hybrids	CD47-expressing tumor exosome–cRGD liposome hybrid (miR497/TP-HENPs)	Sonication/extrusion followed by calcium-phosphate surface assembly and dual-cargo loading (multistep)	miR-497 EE 72 ± 5%; triptolide EE 78 ± 3%	125 ± 6 nm; PDI < 0.2 during 7-day stability testing	cRGD functionalization and CD47 expression; quantitative tumor-accumulation and carrier half-life data NR	Ovarian cancer; final tumor volume 107 ± 27 mm^3^ and tumor inhibition 87% for the complete combination	[38]
EV–liposome fusion hybrids	Folate-modified exosome–lipid hybrid nanoparticles (ALKBH5-ELNPs)	Folate-modified LNP preparation followed by three freeze–thaw cycles with exosomes	*ALKBH5 mRNA*; EE 89%	235 ± 4 nm; PDI 0.24 ± 0.02	ELNPs entered CRC cells and produced higher ALKBH5 expression than ALKBH5-LNPs; luciferase expression localized mainly to colon or subcutaneous tumors at 24–120 h; carrier half-life NR.	Colorectal cancer; restored ALKBH5 expression and reduced tumor size and number; numeric inhibition rate NR	[39]
EV–liposome fusion hybrids	DR5-scFv-engineered exosome–liposome hybrid nanoparticles (TP-siRC@tHyNPs)	Engineered DR5-scFv EVs combined with preloaded liposomes through three freeze–thaw cycles (multistep dual-cargo process)	*CYP3A4 siRNA* plus triptolide; Triptolide EE 80.07 ± 1.18% and drug loading (DL) 7.77 ± 0.16%; siRNA loading metric NR.	Pre-fusion TP-siRC@Lip: 135.77 ± 2.95 nm; final DR5-scFv functionalized hybrid: approximately 150 nm; PDI NR.	Greater tumor accumulation than the hybrid lacking DR5-scFv modification; triptolide half-life 58-fold versus free drug	Melanoma; CYP3A4 inhibition 51.84 ± 6.55%; tumor inhibition 94.91 ± 2.01% in PMM and 91.00 ± 3.83% in PDX models	[40]
EV–liposome fusion hybrids	M1-macrophage exosome–photothermal liposome hybrid (S+G@ELP)	PEG-mediated fusion of M1 EVs and photothermal liposomes followed by dual loading (multistep)	*SerpinB9 *siRNA EE 49.9 ± 3.8%; granzyme B EE 36.3 ± 4.3%; cypate included for photothermal activation	~158.97 nm; PDI NR	Longer blood persistence and greater tumor fluorescence than comparator formulations; numeric half-life and tumor-accumulation ratio NR	B16F10 melanoma; the complete combination suppressed tumor growth; numeric inhibition rate NR	[41]
EV–liposome fusion hybrids	M1-macrophage exosome–cationic photothermal liposome hybrid (si@PLE)	Liposome preparation, three freeze–thaw fusion cycles, followed by siRNA incubation for 15 min and sonication for 10 min (multistep)	*IGF2BP2 *siRNA plus cypate; siRNA EE 89.56% and LE 66.98%	203.8 ± 5.51 nm; PDI 0.437	Greater tumor fluorescence than a composition-matched non-fused liposome; numeric carrier half-life NR	HNSCC; *IGF2BP2 *silencing comparable to Lipofectamine 3000; the combination inhibited CAL27 and FADU xenografts; numeric inhibition rate NR	[42]
EV–liposome fusion hybrids	Tumor-exosome/Akkermansia-OMV/cationic-liposome hybrid nanovesicles (Lipo-PD-L1@HEV)	Cationic liposome complexed with plasmid at an N/P ratio of 5 for 15 min, then sonicated with tumor-cell exosomes and Akkermansia OMVs and co-extruded at least five times through 800-, 400-, and 200-nm membranes (multistep three-vesicle formulation)	PD-L1-trap plasmid DNA, not RNA; in vivo plasmid dose 20 μg; numeric EE/LE NR	Final plasmid-loaded hybrid 170.7 ± 1.9 nm by NTA; ζ 41.2 ± 6.0 mV; PDI NR	After peritumoral administration, signal was mainly localized in the tumor with a small fraction in lymph nodes at 48 h; carrier circulation time NR	B16F10 melanoma and 4T1 breast cancer; reduced tumor growth versus Lipo-PD-L1; 25% of treated 4T1-bearing mice survived >50 days; numeric inhibition rate NR	[43]
EV–liposome fusion hybrids	Irradiation-conditioned tumor exosome–Lipofectamine hybrid (pIL-12@cExo-Lip)	Exosomes from B16F10 cells exposed ex vivo to 20 Gy were fused with Lipofectamine 3000 by low-temperature sonication for 10 min; plasmid complexation for 10 min was followed by 100-kDa ultrafiltration (multistep)	IL-12 plasmid DNA, not RNA; pEGFP used for loading assessment showed EE 83.19%; numeric pIL-12 EE NR	cExo-Lip before pIL-12 loading: 135 ± 5 nm; ζ −2.59 mV; PDI NR	Greater uptake by B16F10 than 3T3 cells in vitro; in vivo biodistribution and carrier circulation time NR	B16F10 melanoma; pIL-12@cExo-Lip plus 6 Gy radiotherapy achieved 91.07% tumor suppression and increased antitumor immune-cell responses	[44]
EV–liposome fusion hybrids	Honeysuckle-derived vesicle-like particle–paclitaxel liposome hybrid (HEV)	Honeysuckle vesicle-like nanoparticles isolated by sucrose-gradient ultracentrifugation and fused with paclitaxel-loaded liposomes by PEG-mediated fusion at a liposome-to-HDVN ratio of 1:3 (multistep)	Paclitaxel LE 13.42 ± 0.06% and EE 86.58 ± 0.06%; endogenous plant miRNAs, including miRNA2911; no exogenously loaded therapeutic RNA	151.13 ± 2.25 nm by DLS (~152 nm by NTA); PDI 0.1904 ± 0.0017 as reported; ζ −13.00 ± 2.25 mV	Intranasal administration produced stronger brain fluorescence for HDVN and HEV than for paclitaxel liposomes; strongest ex vivo fluorescence was in the brain; quantitative biodistribution ratio and carrier half-life NR	C6 glioma; HEV IC50 3.93 μg/mL in vitro; slowed intracranial tumor growth and extended median survival from 21 to 66 days, with lower systemic toxicity than free paclitaxel	[45]
EV–lipid nanoparticle hybrids	Cancer-cell exosome–LNP hybrid nanoparticles (ELNs)	Freeze–thaw or sonication after LNP preparation, with optimization across EV sources (multistep)	*siCD24*, *siCD44,* and *siCD47*; siRNA EE 86.6–89.5% for optimized freeze–thaw hybrids	Optimized freeze–thaw hybrids: DLS 119.3–148.0 nm; NTA 102.5–112.5 nm; PDI 0.16–0.29	~2.5-fold greater parental-cell siRNA association than LNPs and a higher tumor-to-liver fluorescence ratio; carrier half-life NR	Breast cancer and melanoma; >80% reduction of CD24, CD44, or CD47 at 30 nM and reduced tumor growth versus LNPs; numeric inhibition rate NR	[14]
EV–lipid nanoparticle hybrids	Pan02-exosome/LNP hybrid co-delivery system (IGEL)	Microfluidic co-formulation of gemcitabine and circRNA followed by EV–LNP extrusion; losartan preconditioning in vivo (complex multistage regimen)	IL-12 circular RNA plus gemcitabine; numeric circRNA EE/LE NR	125.7 ± 6.52 nm after cargo loading; PDI described as narrow, but numeric value NR	Carrier blood half-life 9.67 h versus 4.88 h for non-EV IGL; tumor signal peaked at 16 h and persisted to 32 h with losartan	Pancreatic cancer; LIGEL produced 46.2% tumor-volume inhibition at day 24; result includes losartan preconditioning and dual cargo	[46]
EV membrane-integrated lipid nanocarriers	Tumor-derived EV membrane–phospholipid hybrid nanovesicles (LEVs)	EV-membrane isolation, thin-film reassembly, extrusion, electroporation, and gel filtration (multistep)	*siCDK1*; siRNA loading up to 56%	117.2 nm; ζ −25.9 ± 0.6 mV; PDI NR	Elimination half-life 44.3 h; tumor fluorescence 2.1-fold versus liposomes at 8 h	Hepatocellular carcinoma; 86.7% *CDK1 knockdown* in Sk-hep1 cells; tumor *CDK1 mRNA* was reduced to 21% of the control level in vivo	[47]
EV membrane-integrated lipid nanocarriers	Phosphatidylcholine-engineered reticulocyte exosomes (PC-Exos)	Post-isolation phosphatidylcholine insertion by incubation and purification (comparatively short process)	Cholesterol-conjugated anti-miR-21; loading did not differ significantly from native EVs; numeric loading value NR	~118 nm; PDI NR	Maximum cellular uptake 1.95-fold in U87 and 1.54-fold in MDA-MB-231 cells versus native EVs; anti-miR-21 fluorescence ~3-fold in U87 cells; no in vivo pharmacokinetic study	Glioblastoma and breast cancer cells; miR-21 silencing 68.4% and VEGF reduction 65% in U87 cells; no in vivo study	[48]

**Abbreviations:** DLS, dynamic light scattering; EE, encapsulation efficiency; EV, extracellular vesicle; HDVN, honeysuckle-derived vesicle-like nanoparticle; HNSCC, head and neck squamous cell carcinoma; LE, loading efficiency; LNP, lipid nanoparticle; NR, not reported; NTA, nanoparticle tracking analysis; OMV, outer-membrane vesicle; PDI, polydispersity index.

**Table 3:** Evidence framework for evaluating functional RNA delivery by EV–lipid hybrid systems.

Evaluation Stage	What Should Be Demonstrated	Representative Measurements	Recommended Controls	Interpretation
Hybrid formation	EV-derived and synthetic components coexist in the same purified particle; complete fusion should be distinguished from mixing, coating, adsorption, and lipid exchange	Post-process purification; single-particle dual-label analysis; cryo-TEM/cryo-ET or AFM; super-resolution imaging; FRET as a lipid-mixing assay; proteomic or topology analysis	EV alone; lipid carrier alone; unfused physical mixture; dye-only and lipid-exchange controls	Bulk colocalization or FRET does not establish that most particles are completely fused
RNA loading and protection	RNA remains associated with the purified hybrid and is protected from extracellular degradation	RNA content; loading or association efficiency; RNase protection; removal of free RNA	Free RNA; parental RNA-loaded carrier; post-purification blank	Measurements should distinguish encapsulated RNA, surface-bound RNA, and residual free cargo.
Cellular uptake	Hybrid-associated RNA reaches recipient cells	Flow cytometry; confocal imaging; quantitative cellular association	Dye-only control; EV and lipid parents; physical mixture; uptake inhibitors where appropriate	Increased particle fluorescence demonstrates cellular association but not cytosolic RNA delivery.
Intracellular transport and cytosolic release	RNA separates from the carrier and reaches the cytosol or another required intracellular site	Separate labeling of membrane and RNA; organelle colocalization; cytosolic delivery or fusion reporters	Membrane-only label; free dye; endosomal controls; matched parental carriers	Reduced lysosomal colocalization or membrane mixing is supportive but should be complemented by cargo-specific evidence.
Functional RNA activity	The delivered RNA retains sequence- and cargo-specific biological activity	Target mRNA or protein knockdown; reporter silencing; protein expression; pathway-specific readouts	Scrambled RNA; cargo-free hybrid; parental carriers; non-target transcript or protein	Gene silencing or protein expression provides the clearest evidence of productive RNA delivery.
Therapeutic response and attribution	The molecular effect contributes to the observed antitumor response	Cell viability; tumor growth; survival; histology; immune or metabolic endpoints	Single cargo components; RNA-free hybrid; free drug; parental carriers; complete formulation	Tumor inhibition alone cannot identify the contribution of RNA, hybridization, or co-delivered treatment.

Abbreviations: EV, extracellular vesicle; RNA, ribonucleic acid; FRET, Förster resonance energy transfer; RNase, ribonuclease. Author-developed framework based on references [10,53,54,55,56].

## Data Availability

No new data were created or analyzed in this study. Data sharing is not applicable to this article.

## Abbreviations

The following abbreviations are used in this manuscript:

AF4	asymmetric-flow field-flow fractionation
AFM	atomic force microscopy
AKT	protein kinase B
ALKBH5	AlkB homolog 5
AMMS	Academy of Military Medical Sciences
AOM/DSS	azoxymethane/dextran sulfate sodium
CaP	calcium phosphate
CD	cluster of differentiation (CD9, CD24, CD44, CD47, CD63, and CD81)
*CDK1*	cyclin-dependent kinase 1
circRNA	circular RNA
cRGD	cyclic arginine–glycine–aspartic acid
cryo-EM	cryogenic electron microscopy
cryo-ET	cryogenic electron tomography
CYP3A4	cytochrome P450 3A4
DLS	dynamic light scattering
DNA	deoxyribonucleic acid
DOPE	1,2-dioleoyl-sn-glycero-3-phosphoethanolamine
DOTAP	1,2-dioleoyl-3-trimethylammonium-propane
DPPC	1,2-dipalmitoyl-sn-glycero-3-phosphocholine
DR5	death receptor 5
DSPE-PEG2000	1,2-distearoyl-sn-glycero-3-phosphoethanolamine–polyethylene glycol 2000
EE	encapsulation efficiency
ESCRT	endosomal sorting complex required for transport
EV	extracellular vesicle
FRET	Förster resonance energy transfer
GMP	good manufacturing practice
HNSCC	head and neck squamous cell carcinoma
*IGF2BP2*	insulin-like growth factor 2 mRNA-binding protein 2
IgG	immunoglobulin G
IL-12	interleukin-12
IL-6	interleukin-6
ISEV	International Society for Extracellular Vesicles
JMJD8	Jumonji domain-containing protein 8
LNP	lipid nanoparticle
M1	classically activated macrophage phenotype
m6A	N6-methyladenosine
MHC I	major histocompatibility complex class I
miRNA	microRNA
MISEV	Minimal Information for Studies of Extracellular Vesicles
mRNA	messenger RNA
mTOR	mechanistic target of rapamycin
NRF2	nuclear factor erythroid 2-related factor 2
NTA	nanoparticle tracking analysis
OMV	outer-membrane vesicle
PD-L1	programmed death-ligand 1
PDI	polydispersity index
PDX	patient-derived xenograft
PEG	polyethylene glycol
PI3K	phosphoinositide 3-kinase
PKM2	pyruvate kinase M2
PMM	pulmonary metastatic melanoma
RNA	ribonucleic acid
RNase	ribonuclease
ROS	reactive oxygen species
scFv	single-chain variable fragment
SEC	size-exclusion chromatography
siRNA	small interfering RNA
TEM	transmission electron microscopy
TFF	tangential-flow filtration
TNF-α	tumor necrosis factor alpha
TP	triptolide
